# Association between daytime sleepiness and impaired expiratory lung function due to smoking in patients with mild-to-severe OSA: an observational study

**DOI:** 10.1007/s11325-025-03530-w

**Published:** 2025-11-13

**Authors:** Giulia Sartori, Alberto Fantin, Marcello Ferrari, Ernesto Crisafulli

**Affiliations:** 1 Department of Medicine, Respiratory Medicine Unit, University of Verona and Azienda Ospedaliera Universitaria Integrata of Verona, Largo L.A. Scuro, 10, Verona, 37134 Italy; 2 Department of Pulmonology, S. Maria della Misericordia University Hospital, Udine, Italy

**Keywords:** Obstructive sleep apnoea, Impaired expiratory lung function, Daytime sleepiness, Chronic obstructive pulmonary disease, Cardiac autonomic response

## Abstract

**Purpose:**

There are no studies about daytime sleepiness (DS) in patients with obstructive sleep apnea (OSA) and impaired expiratory lung function (IELF) due to the smoking habit. We aimed to evaluate the association between DS and IELF in patients with OSA and look for possible DS-related factors.

**Methods:**

In a prospective study, 220 untreated patients with mild-to-severe OSA were divided into non-smokers (Group 1, *N* = 113), smokers without IELF (Group 2, *N* = 69) and smokers with IELF (Group 3, *N* = 38). Data about anthropometric characteristics, main comorbidities, spirometry, polygraphy, and DS (by Epworth Sleepiness Scale-ESS) were collected.

**Results:**

Compared to other groups, patients in Group 3 had lower mean values and the lowest SpO2 (pulse oximetry oxygen saturation), with a greater ST_90_ (sleep time with SpO_2_ below 90%). In Group 3, DS was lower (ESS median 3) in comparison to Groups 1 (median 6; *p* = 0.043) and 2 (median 6; *p* = 0.005)(Kruskal-Wallis test *p* = 0.021). In Group 3, particularly in patients with moderate-to-severe IELF, there was a significant association between ESS values and nocturnal lowest heart rate (LHR) (r² = 0.436; *p* = 0.001). Finally, two multivariate linear regression-adjusted models, the second considering domiciliary treatment with bronchodilators, confirm the association between the nocturnal LHR and ESS (β = -0.278; *p* < 0.001 and β = -0.260; *p* = 0.001).

**Conclusion:**

Patients with OSA and IELF are less sleepy than OSA patients without it, even if they are smokers. A probable effect of autonomic alteration may explain the lower perception of sleepiness in OSA patients with IELF.

## Introduction

Obstructive sleep apnoea (OSA) is characterised by recurrent sleep-related airflow cessation, causing apnoeic events and producing intermittent hypoxemia, autonomic fluctuation, and sleep fragmentation [1]. Mechanistic pathways increasing cardiovascular risk vary across subgroups of OSA [2]. In patients with OSA, the related hypoxic burden, a sign of nocturnal intermittent hypoxemia [3] and symptom subtypes identifying the severity of sleepiness [4], are associated with increasing cardiovascular risk.

Daytime sleepiness (DS) is a public health issue with heterogeneous causes, generally due to sleep deprivation, poor night-time sleep (fragmentation) or sleep disorders [5]. DS is accompanied by deranged cardiac autonomic control at night [6]. A moderate-severe OSA has been identified as an independent predictor of excessive DS [7]. Although developed in 1991 [8], the Epworth Sleepiness Scale (ESS), a commonly used questionnaire to evaluate patients with DS, remains an important tool for measuring this condition [9].

Functionally, smokers with chronic obstructive pulmonary disease (COPD) have impaired expiratory lung function (IELF), causing specific symptoms such as dyspnoea [10]. Sleep with a related increasing prevalence of DS is impacted in patients with COPD due to concomitant OSA or the presence of nocturnal hypoventilation [11]. The poor sleep quality of patients with COPD may be associated with worse clinical outcomes [12]. In general, OSA is common in COPD patients, reaching a prevalence of almost 30% [13]; both COPD and OSA are associated with overlapped pathophysiological disturbances, including hypoxia and inflammation, contributing to cardiovascular comorbidities [14].

There are no studies about the DS in patients with OSA and coexisting respiratory alterations, such as an IELF due to smoking. Therefore, we aimed to evaluate the association between DS and IELF in patients with OSA. The smoking habit has been considered a confounding factor in different groups because it is independently associated with DS in longitudinal studies [15] and a meta-analysis [16].

## Methods

### Study cohort and groups

From September 2020 to July 2024, we prospectively evaluated subjects admitted for a suspected nocturnal respiratory disorder to a dedicated ambulatory of the Respiratory Medicine Unit of the Azienda Ospedaliera Universitaria Integrata of Verona. Firstly, we excluded subjects with normal polygraphy, patients with OSA who received any treatment (for example, continuous positive airway pressure, CPAP), patients with other nocturnal diseases (Obesity Hypoventilation Syndrome-OHS) [17], and patients for whom data related to respiratory polygraphy were missing or had a poor quality track. Therefore, in only untreated patients with OSA, we evaluated the smoking habit, considering non-smokers and current or former smokers; smoking users, current or former, have been considered if having at least 10 packs/year. In non-smokers undergoing spirometry (this exam was not mandatory for these patients), we excluded patients with IELF, while in current or former smokers, a mandatory requirement was to have a spirometry, excluding moreover patients whose spirometry data were of poor quality. Finally, we considered three study groups: Group 1, OSA non-smokers; Group 2, OSA current or former smokers without IELF; and Group 3, OSA current or former smokers with IELF. The presence of IELF was defined if patients at spirometry had an obstructive pattern, such as COPD patients (forced expiratory volume in the first second-FEV_1_/forced vital capacity-FVC < 0.7) [10] or a preserved ratio impaired spirometry (PRISm) (FEV_1_/FVC ≥ 0.7 but with FEV_1_ < 80% of predicted), as reported in current or former smokers [18]. PRISm has been chosen because it reflects a pre-obstructive condition [19], having in smokers a potential role in progressing to overt COPD [20]; finally, PRISm, compared with normal spirometry, was significantly associated with more significant all-cause mortality [21].

The Hospital’s Ethics Committee approved the study protocol, which was conducted following Good Clinical Practices and the Declaration of Helsinki.

**Fig. 1:**
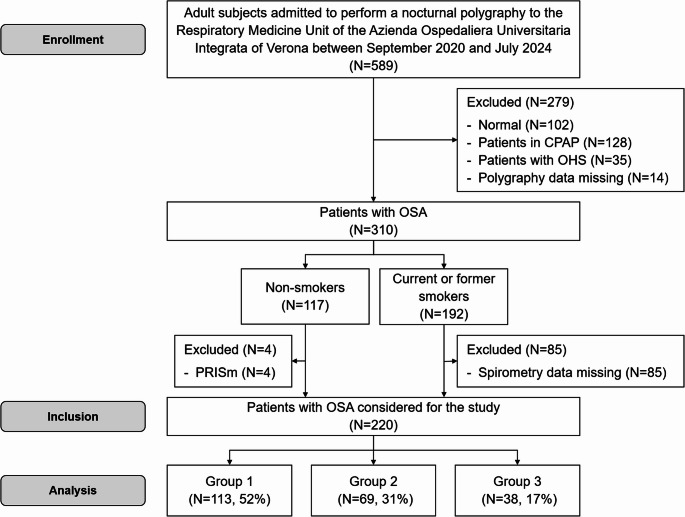
Study flow chart. *CPAP* indicates continuous positive airway pressure, *OHS* obesity hypoventilation syndrome, *OSA* obstructive sleep apnoea, *PRISm* preserved ratio impaired spirometry

Figure 1 shows the study flow diagram.

All subjects were considered eligible for the study upon admission to the dedicated outpatient visit, following evaluation of the inclusion criteria, as determined by respiratory nocturnal polygraphy. Patients were then asked to assess the time required to participate in the research, especially for the daytime sleepiness evaluation. Finally, a written consent form was asked to be signed for all patients who agreed to participate.

### General measures

We collected demographic and anthropometric characteristics, including body mass index (BMI) and body circumferences (neck, waist, and hip). Additionally, data related to the primary comorbidities (arterial hypertension, heart disease, diabetes, peripheral arterial disease, and asthma/COPD) were also collected. Lifestyle information included smoking habits and the related number of packs/year.

COPD was diagnosed according to the 2023 Global Initiative for Chronic Obstructive Lung Disease (GOLD) document [10]. The GOLD 2023 staging considers the events occurring in the preceding year and the dyspnoea perception according to the modified Medical Research Council (mMRC). In detail, stages A and B refer to patients with COPD having an mMRC 0–1 (A) and an mMRC ≥ 2 (B), but both with 0–1 moderate ECOPD (not leading to hospitalisation), while stage E refers to patients with COPD having ≥ 2 moderate ECOPD or at least one ECOPD leading to hospitalisation, independently of the mMRC scale.

### Nocturnal polygraphy and daytime sleepiness

Respiratory polygraphy with a portable Nox T3s™ device (https://noxmedical.com, USA) has been used. The following signals were detected: blood oxygen saturation (measured by a finger sensor), thoracic and abdominal movements (measured by inductive belts), nasal airflow (derived from the belts), snoring and body position (both detected by the device). This portable device has demonstrated excellent measurement agreement compared to in-lab polysomnography [22]. The device’s signals have been analysed using the Noxturnal™ sleep study software, which uses an advanced automated scoring algorithm for respiratory analysis. Each polygraph tracing was subsequently reviewed by a specialist physician, who confirmed the nocturnal respiratory events and the obtained score.

The polygraphy reports analysed according to the American Academy of Sleep Medicine document [23] included the total sleep time in minutes (TST) and the apnoea-hypopnoea index (AHI) calculated as the number of apnoeas and hypopnoeas per hour (n/h). An apnoea was considered as absent airflow for at least 10 s, while a hypopnea was a decrease in airflow (>30%) for at least 10 s associated with oxygen desaturation (≥ 3%). The AHI has also been considered a categorical variable, defining the mild (AHI ≥ 5 and ≤ 15 events/h), moderate (AHI >15 and ≤ 30 events/h), and severe (AHI >30 events/h) stages [1].

Parameters related to pulse oximetry oxygen saturation (SpO_2_) were recorded during polygraphy. In particular, we considered the mean and lowest SpO_2_, the number of events per hour of oxygen desaturation of 3% (ODI), the mean of desaturation, and the sleep time evaluated in minutes with SpO_2_ below 90% (ST_90_) [1]. Among the parameters of nocturnal cardiac response, we considered the mean heart rate (HR), the highest value of HR (HRR), and the lowest value of HR (LHR).

We considered only patients with a recorded polygraphy report with good signal quality.

To evaluate daytime sleepiness, patients completed the Italian version of the Epworth Sleepiness Scale (ESS), a self-report questionnaire designed to assess daytime sleepiness [24]. The ESS consists of eight items that measure the probability of falling asleep during daily life situations, and the total score can range from 0 to 24 [24].

### Lung function measures

Lung function procedures were performed according to international recommendations [25]. A flow-sensing spirometer connected to a computer for data analysis (Jaeger MasterScreen PFT System) was used to measure lung function. FVC, FEV_1_, residual volume (RV) and total lung capacity (TLC) were recorded. FEV_1_/FVC ratio < 0.7 was taken as the index of airflow obstruction [10]. FEV_1_, FVC, RV, and TLC were expressed as percentages of the predicted values [26].

### Specific measures in OSA with impaired expiratory lung function

All these measures, such as lung function measures, have been considered if effectuated in a stable condition and six months from nocturnal polygraphy registration. In the four weeks prior to enrollment, the presence of an intercurrent acute condition characterised by a clinical worsening of perceived dyspnea or of chronic cough with increased volume and purulence of the sputum, or a change in the domiciliary treatment, was considered a criterion of instability [10].

The Italian version of the 5-point mMRC questionnaire was administered to measure the perceived degree of breathlessness related to daily living activities [27]. The mMRC ranges from 0 to 4 and describes the progressive symptoms that may impact an individual with a chronic respiratory condition, from 0, which describes shortness of breath with strenuous exercise, to 4, describing being too breathless to leave the house or breathless when dressing or undressing.

Data from blood gas analyses were also collected concerning pH, partial arterial carbon dioxide pressure (PaCO_2_), and partial arterial oxygen pressure (PaO_2_). Finally, we recorded the prevalence of patients using domiciliary respiratory treatment, either alone or in combination. In particular, we considered the use of long-acting β-agonists (LABA), long-acting muscarinic antagonists (LAMA), and inhaled corticosteroids (ICS).

### Statistical analysis

A preliminary Shapiro-Wilk test was performed to define the distribution of variables in the sample. Data are reported as percentages for categorical variables and mean (with standard deviation-SD) or median (with interquartile range-IQR) for continuous variables. Categorical variables were compared by the χ^2^ test or the Fisher exact test, while the one-way ANOVA with the post hoc multiple Bonferroni comparison and the nonparametric Mann-Whitney U or Kruskal-Wallis H tests assessed continuous variables.

Relationships between variables were assessed using the Pearson (*r* and *r*^*2*^) and the Spearman (ρ) correlation coefficients. In OSA smokers with IELF, a multivariate linear regression model (method: Enter) was performed to identify variables associated with the Epworth Sleepiness Scale, which was considered the dependent variable. Variables that showed a p-value < 0.10 were included in the model. To avoid collinearity, strongly correlated variables (r >|±0.4|) were excluded from the multivariate analyses. Betas (β), standard error (SE), and 95% confidence interval (CI) for β were calculated for each model. Model 1 was adjusted for age, sex, body mass index, presence of heart disease, and diabetes, while Model 2 was adjusted for the variables in Model 1, plus the use of LABA or LAMA.

All analyses were performed using IBM SPSS, version 17.0 (IBM Corp., Armonk, NY, USA), and a p-value of < 0.05 has been considered statistically significant.

## Results

We considered 220 patients with OSA, divided into three groups: Group 1 (*N* = 113, 52%), Group 2 (*N* = 69, 31%) and Group 3 (*N* = 38, 17%). The prevalence of heart disease in Groups 2 and 3 was higher than in Group 1 (45% and 50% vs. 25%, respectively), while Group 3, compared to Group 2, had a higher smoking impact in terms of packs/year. By definition, the worst lung function, as measured by FEV_1_, FVC, and FEV_1_/FVC, was present in Group 3. General and spirometric characteristics are shown in Table 1.

**Table 1:** General and spirometric characteristics of OSA patients

Variables	Group 1 (*N* = 113)	Group 2 (*N* = 69)	Group 3 (*N* = 38)	*p*-value
Age, years	64 [19]	63 [19]	71 [15]	0.107
Male, n (%)	73 (65)	56 (81)	28 (74)	0.053
BMI, kg/m^2^	31.7 [8.9]	31.4 [6.8]	32.4 [8.5]	0.556
Circumferences, cm				
Neck	41 [4]	42 [5]	42 [4.8]	0.207
Waist	109.2 ± 14.8	112.1 ± 13.7	113.3 ± 14.1	0.226
Hip	111 [14]	111 [11.3]	113 [15.8]	0.630
Former/current smokers, n (%)	-	54 (78)/15 (22)	30 (79)/8 (21)	0.934
Packs/year	-	15 [30]	32.5 [29.4]	**0.046**
Asthma, n (%)	11 (9.7)	6 (8.7)	6 (16)	0.485
Arterial hypertension, n (%)	52 (46)	38 (55)	23 (60)	0.229
Heart disease, n (%)	28 (25)	31 (45)^a^	19 (50)^a^	**0.003**
Diabetes, n (%)	17 (15)	17 (25)	12 (32)	0.062
PAD, n (%)	15 (13)	6 (8.7)	3 (7.9)	0.508
FEV_1_, % of predicted	107.8 ± 23.2	107.9 ± 16.9	77.6 ± 23.7^b d^	**< 0.001**
FVC, % of predicted	108.6 ± 23.4	108.5 ± 18.6	90.6 ± 26.6^a d^	**< 0.001**
FEV_1_/FVC, %	79.8 [8.7]	78.2 [5.3]	66.4 [8.7]^b d^	**< 0.001**
TLC, % of predicted	96.3 ± 18.2	98.5 ± 15.9	94.3 ± 17.5	0.533
RV, % of predicted	85.5 [23]	87 [28]	94.5 [35]	0.116

The data are reported as the number of patients (percentage), mean ± standard deviation or medians [interquartiles range]

Percentages are calculated for non-missing data. In bold are reported significant values

Patients were divided into Group 1 (OSA non-smokers without impaired expiratory lung function-IELF), Group 2 (OSA smokers without IELF), and Group 3 (OSA smokers with IELF). Group 3 were patients with chronic obstructive pulmonary disease (COPD) *N* = 27 (71%) and preserved ratio impaired spirometry (PRISm) *N* = 11 (29%)

*OSA* indicates obstructive sleep apnea, *BMI* body mass index, *PAD* peripheral arterial disease, *FEV*_*1*_ forced expiratory volume in the first second, *FVC* forced vital capacity, *TLC* total lung capacity, *RV* residual volume

^a^ and ^b^ represent *p* < 0.05 and *p* < 0.001 versus Group 1; ^c^ and ^d^ represent *p* < 0.05 and *p* < 0.001 versus Group 2

Concerning nocturnal polygraphy data shown in Table 2, Group 3, in comparison to Groups 1 and 2, had worse values of mean SpO_2_ (median 90.6% vs. 92.4% and 92.4%, respectively), lowest SpO_2_ (median 75% vs. 78% and 80%, respectively), and ST_90_ (median 24.3% vs. 8.4% and 9.1%, respectively).

**Table 2:** Nocturnal polygraphy data

Variables	Group 1 (*N* = 113)	Group 2 (*N* = 69)	Group 3 (*N* = 38)	*p*-value
TST, min	463 [37]	461 [33.5]	459.5 [49.8]	0.904
AHI, n/h	18 [21.3]	20.2 [26.6]	14.3 [19.8]	0.176
AHI stages, n (%)				0.601
Mild	48 (42)	26 (38)	20 (52)	
Moderate	36 (32)	21 (30)	9 (24)	
Severe	29 (26)	22 (32)	9 (24)	
Mean SpO_2_, %	92.4 [2.8]	92.4 [2.9]	90.6 [4.5]^b c^	**< 0.001**
Lowest SpO_2_, %	78 [11]	80 [10.5]	75 [17.5]^a c^	**0.031**
ODI, n/h	22.7 [24.5]	23.8 [31]	18.2 [31.7]	0.512
Mean of desaturations, %	5.2 [2.5]	5.2 [3]	5.5 [3]	0.960
ST_90_, %	8.4 [24.2]	9.1 [24.7]	24.3 [50.7]^b d^	**< 0.001**

The data are reported as the number of patients (percentage) or medians [interquartiles range]

Percentages are calculated for non-missing data. In bold are reported significant values

*TST* total sleep time, *AHI* apnoea-hypopnoea index, *SpO*_*2*_ pulse oximetry oxygen saturation, *ODI* oxygen desaturation index, *ST*_*90*_ sleep time with SpO_2_ below 90%

^a^ and ^b^ represent *p* < 0.05 and *p* < 0.001 versus Group 1; ^c^ and ^d^ represent *p* < 0.05 and *p* < 0.001 versus Group 2

The clinical and functional characteristics of patients in Group 3 are shown in Table 3. In general, patients had mild (45%) and moderate (47%) severity of IELF, with a low perception of dyspnea (median mMRC grade 1.5) and normal blood gas analysis values. According to the GOLD 2023 staging, patients with COPD (*N* = 27) were prevalently in stage A (67%), while the higher prevalence of domiciliary treatment used was LABA or LAMA (22%) and triple therapy (ICS + LABA + LAMA, 22%), followed by LABA + ICS (19%). 30% of patients with COPD had no domiciliary treatment.

**Table 3:** Clinical and functional characteristics of patients in group 3 (*N* = 38)

Variables	Value
Severity of IELF, n (%)	
Mild (FEV_1_ ≥ 80% of pred.)	17 (45)
Moderate (FEV_1_ ≥ 50 < 80% of pred.)	18 (47)
Severe (FEV_1_ < 50% of pred.)	3 (8)
mMRC, score	1.5 [1]
pH	7.42 ± 0.03
PaCO_2_, mmHg	44.1 ± 5.7
PaO_2_, mmHg	74.1 ± 9.6
For COPD patients only (*N* = 27)	
GOLD 2023 stages, n (%)	
A	18 (67)
B	5 (18)
E	4 (15)
Domiciliary treatment, n (%)	
No treatment	8 (30)
LABA or LAMA	6 (22)
LABA + LAMA	2 (7)
LABA + ICS	5 (19)
ICS + LABA + LAMA	6 (22)

The data are the number of patients (percentage), mean ± standard deviation or medians [interquartiles range]

*IELF* indicates impaired expiratory lung function, *FEV*_*1*_ forced expiratory volume in the first second, *mMRC* modified Medical Research Council dyspnea scale, *PaCO*_*2*_ partial pressure of arterial carbon dioxide, *PaO*_*2*_ partial arterial oxygen pressure, *COPD* chronic obstructive pulmonary disease, *LABA* long-acting β-agonist, *LAMA* long-acting muscarinic antagonist, *ICS* inhaled corticosteroid.

**Fig. 2:**
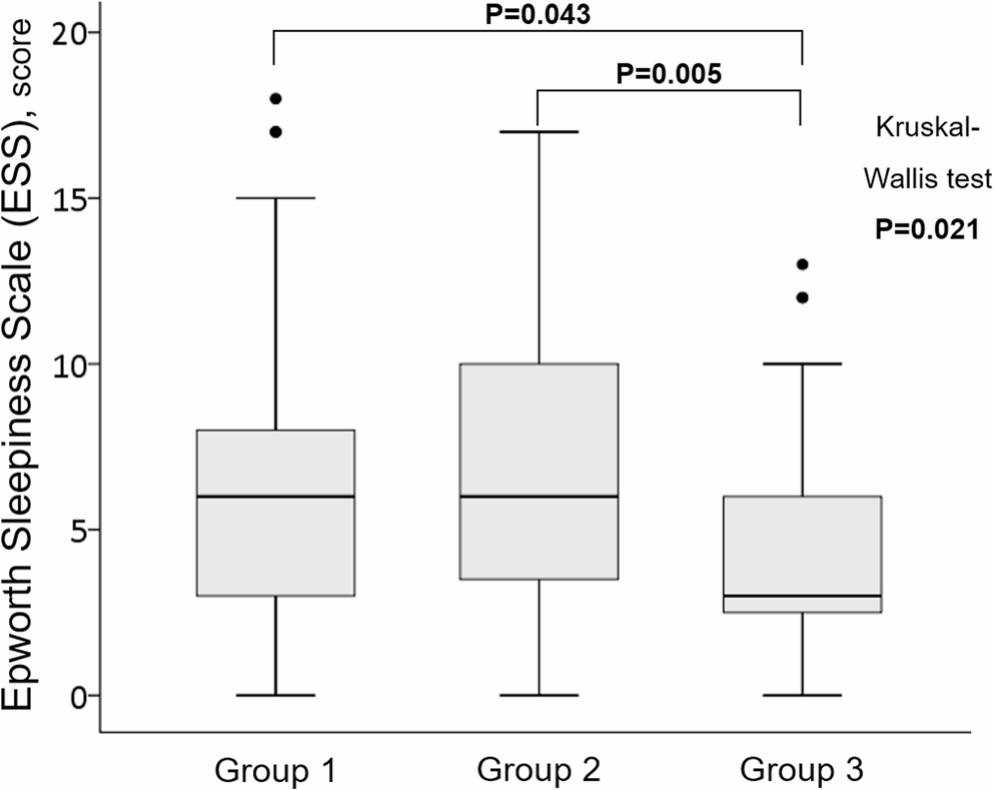
Boxplot of ESS according to the three study groups. *OSA* indicates obstructive sleep apnoea, *ESS* Epworth Sleepiness Scale

In the comparison of ESS (Fig. 2), Group 3 (median 3, IQR 3.75) was lower in comparison to Groups 1 (median 6, IQR 5; *p* = 0.043) and 2 (median 6, IQR 7; *p* = 0.005).

**Fig. 3:**
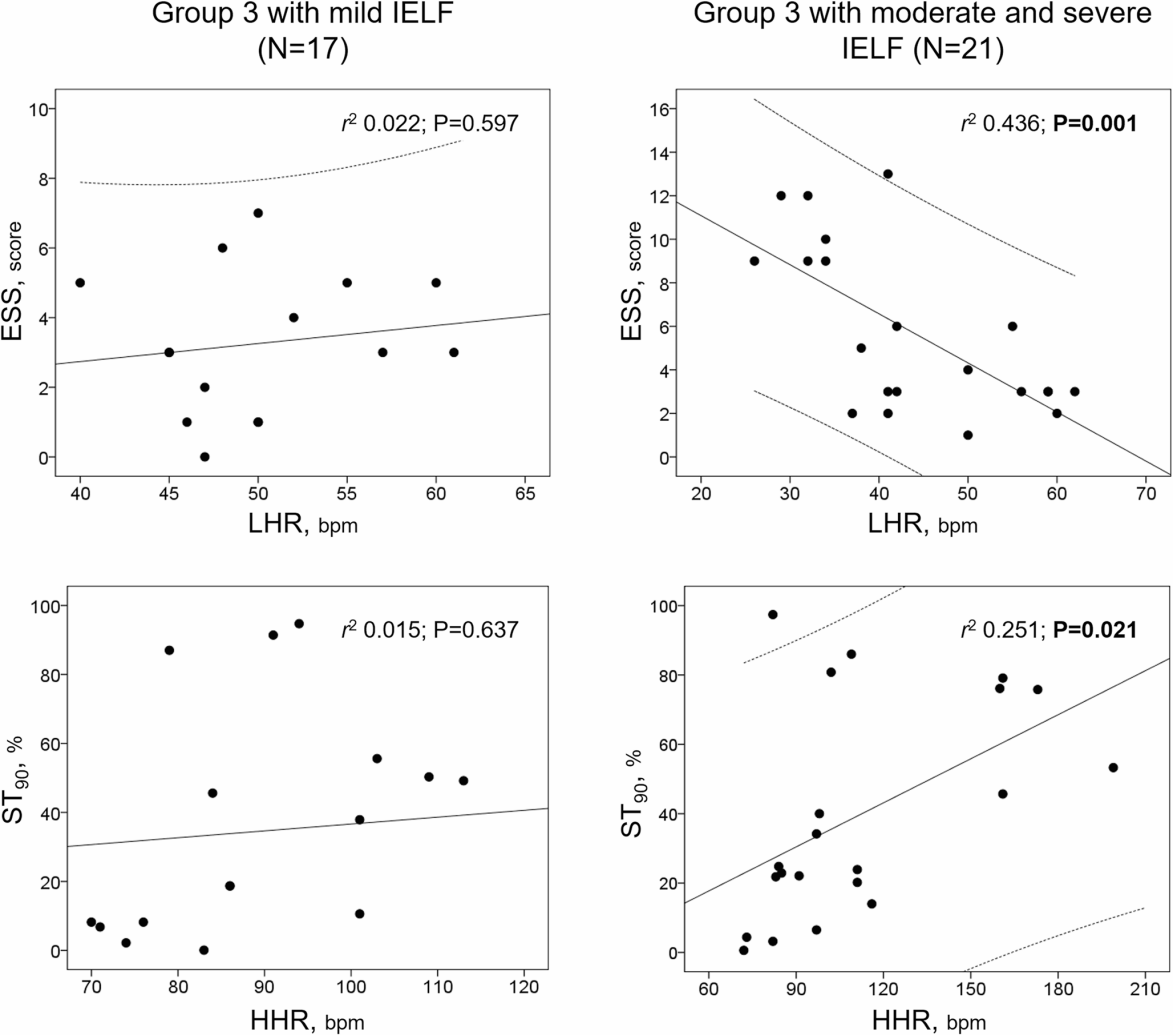
Scatterplots reported according to the severity of impaired expiratory lung function. *OSA* indicates obstructive sleep apnoea, *ESS* Epworth Sleepiness Scale,*ST*_*90*_ sleep time with SpO_2_ below 90%, *HHR* highest heart rate, *LHR* lowest heart rate

Among correlation analyses (Table 4.), in Group 3, ESS was correlated with LHR (ρ −0.430; *p* < 0.01) and with the presence of heart disease (ρ −0.450; *p* < 0.05), while HHR was associated with the lowest SpO_2_ (ρ −0.551; *p* < 0.001), ST_90_ (ρ 0.476; *p* < 0.01), and FEV_1_ (ρ −0.425; *p* < 0.01). No significant correlations were found between ESS and any of the functional lung variables. The correlations between ESS and LHR (*r*^2^ 0.436; *p* = 0.001) and between HHR and ST_90_ (*r*^2^ 0.251; *p* = 0.021) were especially evident in the subgroup of Group 3 patients with moderate to severe IELF (*N* = 21) (Fig. 3). Finally, the linear regression model (Table 5), predicting ESS and performed in Group 3, showed in multivariate analysis adjusted according to the age, sex, body mass index, presence of heart disease and diabetes, a significant association with LHR (β −0.278; 95% CI for β −0.414 to −0.142; *p* < 0.001)(Model 1); this significant association with LHR was confirmed in the adjusted model with the use of LABA or LAMA (β −0.260; 95% CI for β −0.405 to −0.114; *p* = 0.001).

**Table 4.**
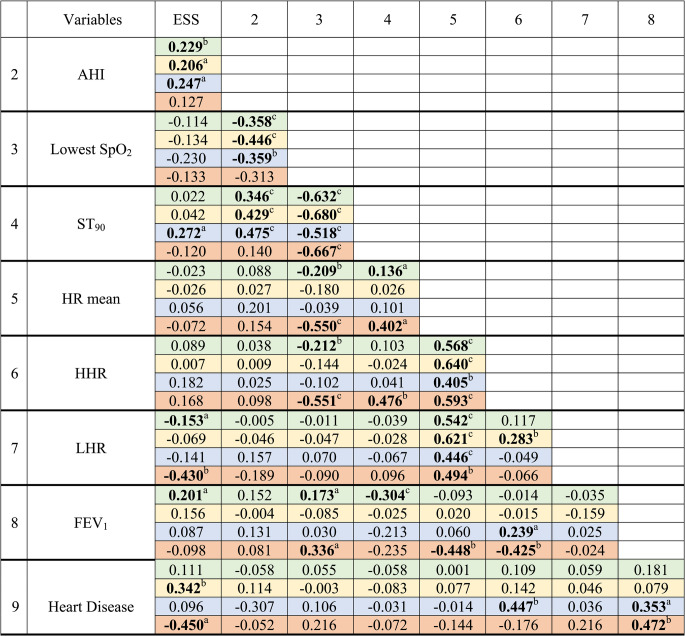
Correlation analyses

Values are reported as Spearman ρ. Bold text indicates a statistically significant difference. Green, yellow, blue, and orange lines represent all patients, Group 1, Group 2, and Group 3. ^a^,^b^ and ^c^ define significant correlation at the 0.05, 0.01 and <0.001 levels, respectively. *ESS* indicates Epworth Sleepiness Scale, *AHI* apnoea-hypopnoea index,*SpO*_*2*_ pulse oximetry oxygen saturation, *ST*_*90*_ sleep time with SpO_2_ below 90%, *HR* heart rate, *HHR* highest heart rate, *LHR* lowest heart rate,*FEV*_*1*_ forced expiratory volume in the first second.

**Table 5:** Multivariate adjusted regression models predicting ESS in patients in group 3 (*N* = 38)

Dependent variable ESS	Multivariate adjusted (Model 1)					Multivariate adjusted (Model 2)				
Independent variables	β	SE	95% CI for β	t	p value	β	SE	95% CI for β	t	p value
AHI	0.020	0.027	−0.034 to 0.075	0.76	0.456	0.036	0.029	−0.024 to 0.096	1.25	0.223
LHR	−0.278	0.066	−0.414 to −0.142	−4.20	**< 0.001**	−0.260	0.071	−0.405 to −0.114	−3.68	**0.001**
	*r* = 0.719; *r*^2^ = 0.517; SE = 2.64; *p* = 0.003					*r* = 0.752; *r*^2^ = 0.566; SE = 2.69; *p* = 0.007				

Bold text indicates a statistically significant difference

Model 1 is adjusted according to the age, sex, body mass index, presence of heart disease and diabetes, while Model 2 is adjusted according to variables in Model 1 plus the use of LABA (long-acting β-agonist) or LAMA (long-acting muscarinic antagonist)

*ESS* indicates Epworth Sleepiness Scale, *AHI* apnoea-hypopnoea index, *LHR* lowest heart rate.

## Discussion

In patients with OSA, the DS is a significant measure of the disease state [5]. Our study demonstrates that patients with OSA and coexisting impaired expiratory lung function (IELF) due to smoking have a selective and low daytime sleepiness perception, not dependent on smoking habits. The nocturnal lowest value of HR (LHR), higher in patients with OSA and IELF, is closely and selectively associated with a low sleepiness perception. This aspect is especially evident in patients with moderate to severe IELF and may be a sign of altered autonomic activity.

Patients with OSA exhibit alterations in the autonomic nervous system (ANS), particularly sympathetic overactivity and reduced parasympathetic activity [28]; these altered autonomic functions may explain the pathophysiology of increased cardiovascular risk [29] and may be modulated in terms of reduced sympathetic activity by an intervention, such as CPAP [30]. Similarly, patients with COPD have cardiac ANS alterations related to increased airway resistance, increased work of breathing, and muscle wasting [31], documented by a depressed variability response to sympathetic and vagal stimuli [32]. This imbalance (increased sympathetic and decreased parasympathetic tone) may promote bronchodilation [32] and is closely associated with ventilatory response during incremental maximal exercise [33].

DS is defined as the inability to stay awake and alert during major waking episodes, although the clinical presentation of DS can vary between patients, including a continuous state of persistent somnolence [23]. When DS is evaluated using a self-report assessment tool, such as the ESS, its prevalence in the general population varies considerably, ranging from 8.5% to 22% [15]. In patients with OSA, DS is related to key factors, including the degree of sleep fragmentation, arousals and the distribution of sleep stages, as well as the frequency of apneas and hypopneas and the degree of overnight oxygen desaturation [1, 5]. Additionally, the alteration in cardiac autonomic control at night may be related to excessive daytime somnolence [6]. This phenomenon is present in college students when several components of sleep (subjective sleep quality, sleep latency, and sleep medication) are associated with autonomic functioning in terms of greater sympathetic dominance [34].

Patients with COPD and OSA generally report a higher level of sleepiness perception than those without OSA [13, 35]. However, our findings, considering also functional conditions related to IELF such as PRISm [18] and smoking habit as a confounder by a specific group [15, 16], focused for the first time on the problem from another point of view when patients with OSA in which the coexistence of both respiratory conditions (OSA plus IELF), in comparison to OSA without IELF, have a surprisingly lower sleepiness perception (Fig. 2). OSA in COPD patients leads to repeated bouts of upper airway obstruction, accompanied by hypoxemia, which causes repetitive arousals. This likely produces ongoing sympathoexcitation in the awake state, resulting in persistent vasoconstriction [36]. The strong inverse correlation between ESS and LHR (Table 4.) and the close association in the two models of the multivariate-adjusted linear regression analysis (Table 5) permit us indirectly to highlight a pathophysiological progressive chronic state of cardiac autonomic alterations with higher nocturnal basal HR, also mediated by hypoventilation and especially in patients with moderate to severe IELF (Fig. 3). The increased double sympathetic activation (for OSA during the night and for IELF during the entire day), but probably reduced parasympathetic activity, typical of patients with chronic airflow obstruction [32, 33], may provoke increased cortical activation and alertness. In this context, the reduced parasympathetic activity may explain the higher LHR values in our OSA patients with IELF. Notably, the primary neural control of bronchiolar diameter is achieved via the parasympathetic nervous system by axons travelling in the vagus nerve, supplying the smooth muscle of the airways [36].

A sympathetic overactivity characterises heart failure (HF), which, although compensatory in the initial phases of the disease, exerts adverse cardiovascular effects with time [37]. In patients with HF and OSA, the subjective daytime sleepiness evaluated by the ESS is inversely related to daytime muscle sympathetic nervous system activity [38]. This relationship is likely mediated via central adrenergic alerting mechanisms [38]. Notably, the ESS was not associated with respiratory variables such as AHI, arousal index, or indices of oxygen desaturation [38]. In addition, in patients with HF and sleep apnea, the degree of subjective daytime sleepiness is inversely related to the mortality risk, suggesting a reduced perception worsening the patient’s prognosis [39]. Our findings on the reduced DS perception in patients with OSA and IELF compared to those without may be seen as a sign of parallelism between COPD [31] and HF [37], two conditions with ANS alterations. In addition, our smokers OSA patients (Group 2, but especially Group 3) also have a higher prevalence of reported heart disease (Table 1), related to the common risk factor of smoking habit and to the synergism between a chronic bronchial condition (IELF) and a cardiac involvement (Group 3). Although in Group 3 the presence of heart disease was associated with DS perception (Table 4.), the multivariate models that also adjusted for it confirm the association between LHR and ESS (Table 5). In any case, patients with two associated conditions (COPD + HF) have a worse prognosis than patients with one single condition [40], similar to OSA with COPD having a worse outcome [11]. Of note, although the Sleep Heart Health Study demonstrates that in patients with OSA, the severity of sleepiness is associated with increasing incident cardiovascular disease, coronary heart disease, and heart failure, with excessively sleepy again increased risk compared with other subtypes [4], in the same study is not considered the coexistence of COPD or in any case a respiratory functional alteration. Therefore, this aspect will be delegated to future studies. Despite this, the ability to identify patients with OSA among COPD patients using the ESS is reduced, especially in advanced stages [41].

### Strengths and limitations

Our strength points are related to the possibility of exploring, for the first time and in a relatively adequate sample of untreated patients with OSA, the role of IELF on daytime sleepiness. As limitations, we considered that although all patients with functional alterations had impaired expiratory flows, a limited number of patients with COPD were included, of whom 30% had no domiciliary treatment. Our choice to also include patients with PRISm, a pre-obstructive condition [19], starts from the consideration of the Copenhagen City Heart Study, in which the BMI was the strongest predictor of FEV_1_/FVC, with an underdiagnosis of airflow obstruction in overweight and obese patients [42], a typical condition associated with OSA. Moreover, not all non-smokers have undergone spirometry so we may have underestimated the prevalence of IELF in Group 1. Secondly, we evaluated the DS with the ESS; however, using a specific questionnaire, such as the COPD and Asthma Sleep Impact Scale (CASIS), would have provided additional information on the association between subjective sleep quality and disease status in our COPD patients [43]. Third, our study’s lower and higher nocturnal HRs represent indirect measures of ANS alterations. Measures of a more solid scientific consideration, such as heart rate variability [44], need to confirm our hypothesis.

## Conclusion

Patients with OSA and coexisting IELF are less sleepy than OSA patients without it, even if they are smokers. A probable effect of autonomic alteration may explain the lower perception of sleepiness in patients with OSA and IELF.

## Data Availability

The datasets used and/or analysed during the current study are available from the corresponding author on reasonable request.

## Declarations

### Clinical trial registration

This study was not registered.
